# Comparing School-Aged Refraction Measurements Using the 2WIN-S Portable Refractor in Relation to Cycloplegic Retinoscopy: A Cross-Sectional Study

**DOI:** 10.1155/2021/6612476

**Published:** 2021-05-21

**Authors:** Ziming Liu, Emmanuel Eric Pazo, Hong Ye, Cui Yu, Ling Xu, Wei He

**Affiliations:** ^1^He Eye Specialist Hospital, Shenyang, China; ^2^Liaoning University of Traditional Chinese Medicine, Shenyang, China; ^3^He University, Shenyang, China

## Abstract

**Purpose:**

To assess the repeatability and agreement of refractive measurements using 2WIN-S photoscreening with the gold-standard cycloplegic retinoscope refraction.

**Design:**

Single centre, cross-sectional study.

**Methods:**

Spherical, cylindrical, axis, and spherical equivalent of 194 bilateral eyes of 97 children were assessed using a retinoscope and 2WIN-S. One week later, another operator repeated the 2WIN-S measurements. The primary outcome measures were to assess the repeatability and agreement between spherical equivalent, J0, and J45 readings of 2WIN-S. The repeatability of measurements was assessed by the within-subject standard deviation (2.77 Sw) and intraclass correlation coefficient (ICC). The agreement between devices was assessed using 95% limits of agreement. The extent of the agreement between cycloplegic retinoscopy and noncycloplegic 2WIN-S measurements was assessed using Bland–Altman analysis.

**Results:**

The mean age ± SD was 10.3 ± 2.46 year (range, 4–14 years). The sphere, cylinder, and spherical equivalent measurements were found to be consistent with both apparatus (*r* value >0.86). ICC for SE, J0, and J45 was 0.900, 0.666, and 0.639, respectively; Sw for SE, J0, and J45 was 0.61D, 0.30D, and 0.31D, respectively; Bland–Altman analysis of retinoscopy with cycloplegia and 2WIN-S for SE was 184/194 (95%) in 95% confidence interval, and the mean value was 0.46. J0 was 184/194 (95%), and the mean value is −0.04. J45 was 181/194 (93%), and the mean value is −0.15.

**Conclusion:**

The objective refractive measurement of 2WIN-S had good reliability and high agreement with the gold-standard retinoscopy refraction in children and adolescents. While consistency was observed, it is essential to take into consideration that it is a screening tool.

## 1. Introduction

**Figure 1:**
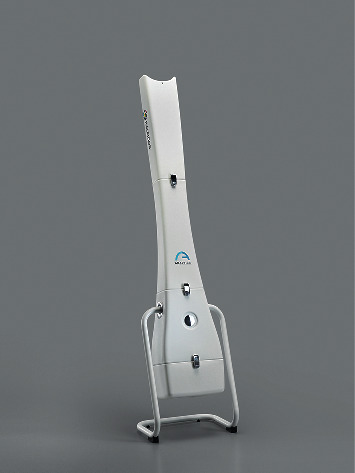
The 2WIN-S includes a photorefractometer (2WIN) and an occluder tube.

Globally, uncorrected refractive errors are the leading cause for moderate to severe vision impairment and the second most common cause for blindness [1]. At present myopia is reaching epidemic proportions in East Asia [2] and is predicted to be prevalent in nearly half of the world's population by the year 2050 [3]. Even though refractive errors can generally be corrected with glasses, contact lenses, or refractive surgery, according to Pascolini et al., uncorrected refractive errors remains the primary reason for visual impairment in 43% of the world population due to the lack of availability and affordable screening for refractive correction [4]. Amblyopia along with refractive errors, strabismus, and anisometropia are reported to be the most common amblyopia risk factors [5]. Additionally, amblyopia treatment is limited by age. Therefore, early screening and diagnosis are paramount in the prevention of amblyopia [6]. Traditional vision screening methods in children can be difficult due to poor cooperation and labour-intensive procedure, and therefore, a handheld photorefractometer offers reduced assessment time in detecting refractive errors. Photoscreening devices are a screening tool to assess refractive error and thereby rule out amblyogenic ametropias in children [7]. It uses infrared light and camera to assess the correct alignment of the red reflex in both undilated eyes and estimates the refractive error, and then, noncycloplegic refractive status, pupil size, and gaze deviation is also calculated [8]. Various photoscreening devices have been recommended by the American Academy of Paediatrics, the American Academy of Ophthalmology, the American Association for Paediatric Ophthalmology and Strabismus (AAPOS), and the American Association of Certified Orthoptists for amblyopia detection in children [9]. Effectiveness of photorefractometer devices has been tested for detection of anisometropia, hyperopia, myopia, and astigmatism [10]. Portable design and instantaneous assessment by looking at the sensor of handheld photoscreening devices makes it a convenient tool for testing children younger than three years and with developmental disorders. However, autorefractors can overestimate myopia and underestimate hypermetropia [11]. Obtaining accurate refractive error measurements in young children is a challenging exercise. Traditionally, the retinoscope has been used to obtain an objective measurement of refraction, and in well-trained hands, it is still considered a very accurate and effective method [12]. Refracting young children, retinoscopy (often combined with cycloplegia) remains the method of choice in most clinical practices. Nonetheless, retinoscopy can be user-dependent, interobserver variability, less accurate in high ametropia, and measured in increments of only 0.25D [13]. The main advantage of 2WIN-S and other handheld photorefractometers is their portability and an approximate 1 m working distance. Binocular measurements are simultaneously obtained in a short amount of time along with pupil diameter [11]. The sensitivity and specificity of 2WIN (Adaptica, Padua, Italy) have previously been validated in detecting ARFs [14–16]. 2WIN along with its recently developed “special light occlude tube” is known as 2WIN-S (Figure 1), which blocks visible light and helps transmit infrared within the chamber.

In this study, we performed refractive error vision screening on 4-year to 14-year-old children in a Chinese population using both 2WIN-S and cycloplegic retinoscopy. Reliability, reputability, and agreement of refractive measurements using the 2WIN-S in relation to cycloplegic retinoscopy refraction were evaluated as cycloplegic retinoscopy refraction is the gold standard for assessing refractive errors [17].

## 2. Methods

### 2.1. Subject Population

The study was approved by the Research Ethics Committee of the He Eye Specialist Hospital. All procedures were reviewed and approved by the local institutional ethical review board in accordance with the Declaration of Helsinki principles. Written informed consent was obtained from all parents. Inclusion criteria were age 4–14 years of age with a corrected visual acuity of 0.1 logMAR (logarithm of the minimum angle of resolution) (6/7.5) or better and were recruited from September 2019 to December 2019. Exclusion criteria were ocular pathology known to interfere with autorefractor performance and abnormalities such as strabismus and previous ocular surgery. Bilateral eyes of subjects were included in the study. Consecutive patients were enrolled in this study when both eyes fulfilled the inclusion and exclusion criteria. Participants were excluded when only one eye fulfilled the inclusion criteria. A total of 194 eyes of 97 subjects (47 male and 50 female) aged 4–14 were assessed using the 2WIN-S refractometer (software version 24.0) and retinoscope (YZ6H; 66 Vision Corp., Suzhou, China). A minimum sample of 150 participants was set with a minimum power of 90% at a 5% level of significance to detect a paired difference of at least 0.75 ± 1.25D–0.3 ± 0.5D.

Initially, noncycloplegic refraction was performed with a retinoscope by a trained ophthalmologist (CY); then, immediately, three photorefractions were carried out by 3 experienced ophthalmologists (LZM, EEP, and HY) using the 2WIN-S, so that the measurements and results were not influenced by operator bias. Then, an objective refraction was performed by a trained ophthalmologist (CY) (who was not informed of the results of the refraction results from the 2WIN-S) after instilling one drop of 1% cyclopentolate (Alcon), and a second drop was administered after 10 minutes; cycloplegic retinoscopy refraction was performed after the waiting period of 30 minutes (40 minutes in total). To assess the repeatability of the 2WIN-S, three consecutive refractions were taken for each participant in the study. The mean value of the three measurements was used in the final analysis and comparison. All measurements were taken between 10 am and 5 pm to minimize the effect of diurnal variation on refraction. To minimize bias, all examiners were masked to the results of other refractive measurements and were conducted in separate rooms.

### 2.2. Objective Refraction

Dry retinoscopy and cycloretinoscopy were performed once in each eye. Retinoscopy was performed in each eye over phoropter lenses, attempting to refine the retinoscopy to be within a ±0.25 D range over the real power for both the spherical and the cylindrical components and the axis to ±5° maximum error. The spherical power, cylindrical power, and cylinder axis displays on the phoropter were covered, so the examiner could not see them. These measurements were performed first without cycloplegia and repeated 30 min later with cycloplegia. This was performed by instillation of one eyedrop 1% cyclopentolate in each eye twice with a 10-minute interval.

### 2.3. 2WIN-S Refractor

The 2WIN photorefractometer can carry out binocular or monocular measurements (Figure 1). The spherical measurement range is between–15.00D and 15.00D at 0.25D step, and cylinder measurement range is between −5.00D and +5.00D at 0.25D step. The measurement results are indicated as red (unreliable) and green (reliable). The 2WIN results outside the measurement range are indicated as “hyperopia” or “myopia.” If, after several attempts, the device was unable to obtain a picture to provide a computer printout result, the tester made the notation “unable to obtain a reading.” The 2WIN-S with an updated software version (Software version 5.3) function combines the photorefractometer with a occlude tube that can block ambient light and helps in the transmission of infrared light utilized by the 2WIN-S photorefractometer as it serves as a darkroom and allows the exam to be performed in any light condition. While the patient looks inside of it, the system automatically detects refractive errors in less than three seconds. 2WIN-S device can concurrently measure the refraction, corneal reflex, pupil size, and interpupillary distance. On occasions, when the device was unable to obtain a measurement to provide a result, the tester made the notation, “unable to obtain reading.” And these participants were removed from the final assessment (*n* = 11 participants). The examiner requested the subjects to look at the bottom of the 2WIN-S tube with wide-open eyes. Since the photorefractometer was placed inside the occlude tube, the user interface screen was operated by a computer tablet which relayed real time screen of the 2WIN-S. Two green circles and a horizontal line appear around the patient pupils. As advised by the manufacturer, measurements were only recorded if they had a reliability index higher than 5 (maximum is 9); measures were repeated when the reliability index was 6 or less (maximum is 9). Eleven participants' pupils were obstructed due to eyelashes and eyelids while performing the 2WIN-S assessment, and their measurements were not captured. The eyelids of these participants had to be manually helped open by the clinician. Additionally, there were 5 participants who did not cooperate while having their eyes assessed with the 2WIN-S and were excluded in the study and analysis. All personal identifiable data of individual participants were deleted (if considered not necessary for this study), coded, and anonymized during or after data collection.

### 2.4. Statistical Analysis

Refractive errors (spherical (S), cylinder ©, axis (A)) were measured five times by retinoscopy in each eye, and the mean vector value was calculated as the final result. All results were converted into power vectors (SE, J0, and J45) as described by Thibos et al. [18] Spherical equivalent (SE) and vector presentation of astigmatism J0 and J45 were calculated according to the following formulas: **SE** = *S* + C/2; **J0** = (−C/2)^*∗*^cos (2^*∗*^*ϑ*); and **J45** = (−C/2)^*∗*^sin (2^*∗*^*ϑ*), respectively [18]. The statistical analyses were performed with commercial software (SPSS ver. 25.0; SPSS Inc.). First, the data were checked for normality using the Kolmogorov–Smirnov test. If the significance value of the test was below 0.05, the data were assumed to have a nonnormal distribution. Since the continuous variables in this study were not normally distributed, they were presented as median and range (minimum value and maximum value). Categorical variables are presented as numbers and frequencies. Frequencies were compared using Pearson's chi-square test. Comparisons between the measurements were performed using the Wilcoxon signed-rank test and Spearman's correlation analysis. The correlations were defined as weak if *r* was below 0.3, moderate if *r* was between 0.3 and 0.7, and strong if *r* was higher than 0.7. To determine the intraobserver repeatability, interobserver and intersession reproducibility, and within-subject standard deviation (Sw), finally, the agreement between the refraction measurement methods was investigated via Bland–Altman analysis. *P* value of <0.05 was assumed to indicate statistical significance.

## 3. Results

**Figure 2:**
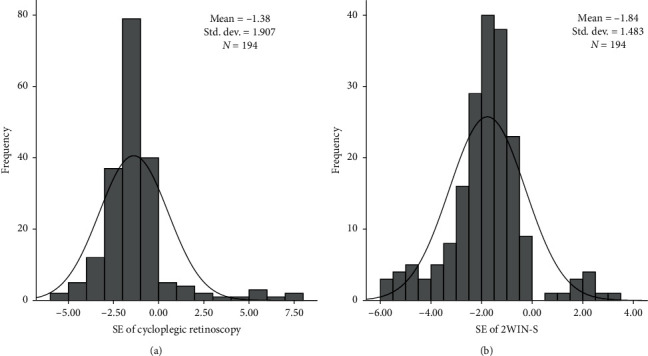
(a) Frequency distribution of spherical equivalent with cycloplegic retinoscopy measurement. (b) Frequency distribution of spherical equivalent with 2WIN-S photorefractometer measurement.

In total, 194 eyes of 97 participants (47 male and 50 female subjects) aged 4–14 (mean 10.3 ± 2.46) years were included and assessed in this study. In total, 16 participants were excluded from the study, of whom 5 were noncooperative and 11 participants pupils were obstructed due to eyelashes and/or eyelids while performing the 2WIN-S assessment.  Table 1 provides the comparative measurements taken with cycloplegic retinoscopy and 2WIN-S. Figures 2(a) and 2(b) show the distribution of spherical equivalent values for cycloplegic retinoscopy and 2WIN-S.

### 3.1. Spherical Findings

The range of spherical results for cycloplegic retinoscopy was −5.75 to +7 D, while the range for those of 2WIN-S was −5.50 to +2.75 D. The mean differences for 2WIN-S minus cycloplegic retinoscopy was −0.39 D. The minus value indicates an underestimation of hyperopia and overestimation of myopia by a photorefractometer when compared with cycloplegic retinoscopy.

### 3.2. Cylindrical Findings

The range of cylindrical results for cycloplegic retinoscopy was −3.25–3.00 D, while the range for 2WIN-S was −3.50–3.00D. The mean differences for 2WIN-S minus cycloplegic retinoscopy was −0.12 D. The minus value indicates an underestimation of hyperopia and overestimation of myopia by a photorefractometer when compared with cycloplegic retinoscopy.

### 3.3. Spherical Equivalent Findings

The range of spherical equivalent results for cycloplegic retinoscopy was −6 to +7.25 D and 2WIN-S was −6.00 to +3.25 D. The mean difference for 2WIN-S minus cycloplegic retinoscopy was −0.45 D. The minus value indicates underestimation of hyperopic spherical equivalent and an overestimation of myopic spherical equivalent by the photorefractometer when compared with cycloplegic retinoscopy.

### 3.4. J0 and J45 Findings

The range of J0 results for cycloplegic retinoscopy was −0.47 to +1.60 D and 2WIN-S was −1.49 to +1.73 D. The mean difference for 2WIN-S minus cycloplegic retinoscopy was −0.30 D. The range of J45 results for cycloplegic retinoscopy was −50 to +0.40 D and 2WIN-S was −0.44 to +0.57 D. The mean difference for 2WIN-S minus cycloplegic retinoscopy was −0.02 D.

### 3.5. Agreement

**Figure 3:**
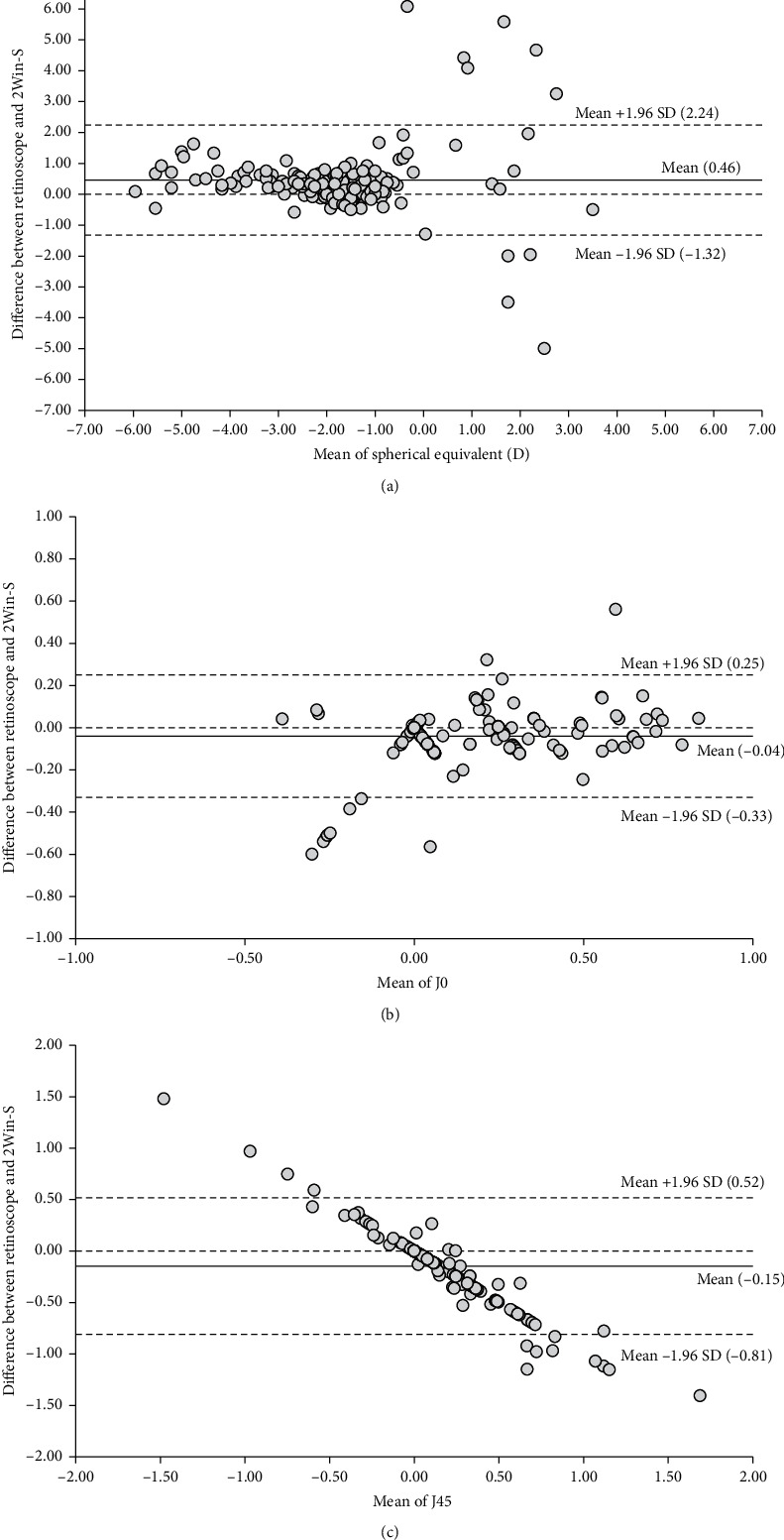
(a) Bland–Altman plot for spherical equivalent. (b) Bland–Altman plot for J0. (c) Bland–Altman plot for J45.

Pearson's correlation test (Table 2) showed a strong and significant correlation between both photorefractometer and cycloplegic retinoscopy measurements. All comparisons showed highly significant correlations. The mean difference for spherical equivalent was 0.46, and 184/194 (95%) eyes were within the 95% confidence interval (Figure 3(a)). The mean difference for J0 was −0.04, and 184/194 (95%) eyes were within the 95% confidence interval (Figure 3(b)). The mean difference for J45 was −0.15, and 181/194 (93%) %) eyes were within the 95% confidence interval (Figure 3(c)).

### 3.6. Reliability and Repeatability

Table 3 provides the repeatability of corneal astigmatism, J0, and J45 measurements by 2WIN-S with regards to cycloplegic retinoscopy. The repeatability of these parameters was good with 2WIN-S, with an ICC for SE, J0, and J45 of 0.90, 0.67, and 0.64, respectively. The 2.77 Sw of repeated corneal astigmatism, J0, and J45 measurements was 0.61D, 0.30D, and 0.31D, respectively.

## 4. Discussion

Reliable measurement of refraction and ocular alignment in children is challenging. The three commercially available infrared photoscreeners, PlusoptiX, SPOT, and 2WIN-S, are designed for paediatric screening. 2WIN-S in a recent 5.3 software release performed similarly to SPOT and slightly less well than PlusoptiX [15]. The purpose of this study was to assess the refraction reliability and repeatability with the 2WIN-S photorefractometer in comparison to a cycloplegic retinoscopy. According to the results, SE was repeatability of 2WIN-S measurement results is very high, and J0 and J45 are reasonable for a screening device.

Objective vision screening is important for children as it helps the clinicians assess visual development; therefore, children from 4 to 14 were included in this study. Gold standard [17] cycloplegic refraction refractive techniques were employed to validate the 2WIN cycloplegic refraction by an experienced. This study compared updated software on the 2WIN and included the occlude tube (2WIN-S) for estimation of noncycloplegic refraction to cycloplegic retinoscopy. We found remarkable comparability of the 2WIN-S to cycloplegic retinoscopy with respect to cylinder power, and both vector components of cylinder are related to axis. Compared to cycloplegic exam spherical equivalent, 2WIN-S had good correlation; however, the slope of the regression curve indicated that 2WIN-S exposed from about 1 meter produced less accommodation. Photoscreening uses a slightly off-lens-axis flash that produce light crescent in the pupillary red reflex. The further the light reflex encroaches in the pupil, the greater the refractive error. For many photoscreeners, the pupillary crescent appears with ocular defocus of >1.5D either hyperopic or myopic. We sometimes observed uninterrupted accurate refraction estimate by 2WIN-S whether outside or within this refractive range which is a typical photoscreening null zone.

Compared to the conventional cycloplegic retinoscopy, various autorefractors such as the 2WIN-S, a variation of the 2WIN refractometer, has indispensable merits for the large-scale vision screening [19, 20]. As examples, it is a portable instrument without connection to a laptop computer, has faster data acquisition, and is patient friendly as the fixation target can be achieved by just looking into the occlude tube (Figure 1). While cycloplegic retinoscopy is time-consuming, uses cycloplegic eye drops, and requires more optometrists, it is not an optimal approach for amblyopia risk factor screening. The new visible-light blocking chamber tube with the 2WIN-S also helps in infrared-transmitting for assessing the refractive error of the subject. It also provides the optimal distance for the device to assess the subject as, unlike other handheld refractometer devices where the assessor might have to move forwards and backwards to get the optimal distance from the patient. Since the 2WIN-S has a chamber to look into, it also eliminates visual distractions for younger subjects while obtaining their refraction. We advised the subjects to place looking part of the chamber against their eyebrow rather than their eyelids. This allowed the chamber and the refractometer inside the chamber to be accurately aligned to the subjects' eyes. 2WIN-S with the chamber gave a faster interpretation of horizontal and vertical alignment and refractive error in a sequence with both eyes open in comparison to 2WIN (without the infrared occluder). The current version of the software estimates large and small values in prism diopters; reliable measurements from 2WIN-S were mainly greater than 10 prism diopters. 2WIN-S, with its near-total dark internal chamber, is useful in extremely photophobic children, such as those with active herpetic keratouveitis as the red reflex can be assessed without employing any visible light [21].

In this study, photorefraction using the 2WIN-S without a cycloplegic agent was compared with cycloplegic retinoscopy in the evaluation of refractive errors in children and adolescent. The findings show that there is a mean difference of -0.39 D between the spherical results of 2WIN-S and those of cycloplegic retinoscopy. This result indicates that myopia tends to be overestimated, and hyperopia tends to be underestimated by the 2WIN-S compared with cycloplegic retinoscopy with a strong 0.88 Pearson's correlation, which indicates a consistent agreement between 2WIN-S and cycloplegic retinoscopy. Bland–Altman analysis of cycloplegic retinoscopy and 2WIN-S demonstrated greater than 93% of the eye assessments were with the 95% confidence interval. Compared with retinoscopy with gold standard (retinoscopy with cycloplegia), a 2WIN-S photorefractometer showed a high degree of consistency in the equivalent spherical and cylinder measurements; therefore, it is safe to mention that it is an effective tool for detecting and screening the diopter of uncooperative patients and screen for refractive error in children.

### 4.1. Limitations

There were limitations on the current version of the 2WIN-S as it has several analogue buttons for various functions and a smaller screen in comparison to other infrared photoscreeners and touchscreen. However, the time required to assess subject are similar to other refractometers [14], and the 2WIN-S solves the several analogue buttons limitation as the app-based system allows the clinician a touch screen tablet size user interface for operations. Although this study had sufficient number of patients including a large number of younger children, the following study limitations were noted: some of the patients were new referrals, but others were already accustomed to wearing their spectacles. Compared to newly referred hyperopic children, consistent spectacle-wear improvement could influence accommodative ability, and therefore, some components of the refractive and alignment values and the percentage of age group from 4 to 6 years of age was 9%. Additionally, in our study, the 2WIN-S was used without cycloplegia and showed a tendency to underestimate hyperopia. This can also be due to accommodation of the eye; therefore, only objective refraction in children can be used as a diagnostic procedure, for example, for prescription of glasses, and can only be performed with cycloplegia. Similar to other automated photoscreeners [22], we strongly suggest that automatic photoscreening like the 2WIN and 2WIN-S should be used with great caution when determining manifest refractions, especially in younger patients in whom accommodation is more active than in elder patients. This is because significant instrument myopia can be induced by the device or the real hyperopia may be unrevealed. A cycloplegic refraction with a retinoscopy is still the gold standard in these eyes and would afford acceptably accurate baseline refractive data as a guideline for clinical prescription. Although manual retinoscopy can be arduous, it can provide an accurate assessment of the refractive error. Future studies on the 2WIN-S would include a larger sample size with higher number of hyperopic eyes and tests its performance in screening amblyopic children.

In conclusion, spherical equivalent, J0, and J45 agreement between 2WIN-S and cycloplegic retinoscopy in this study had high correlation. The portability of 2WIN-S and ease of use in comparison to retinoscopy or table-mounted autorefractometers make it an effective screening tool for large epidemiologic studies, when a high speed is required. It is also ideal in children and disabled people who are not cooperative.

## Figures and Tables

**Table 1:** Comparison of measurements between cycloplegic retinoscopy and 2WIN-S.

	Cycloplegic retinoscopy			2WIN-S			*P* value (paired *t*-test)		95% CI	
	Mean (SD)	Min	Max	Mean (SD)	Min	Max	Δ		Lower	Upper
Spherical (D)	−1.23 (1.78)	−5.75	7.00	−1.62 (1.37)	−5.50	2.75	−0.39	<0.01^*∗*^	−0.52	−0.27
Cylindrical (D)	−0.30 (0.67)	−3.25	3.00	−0.42 (0.68)	−3.50	3.00	−0.12	<0.01^*∗*^	−0.16	−0.07
Spherical equivalent (D)	−1.38 (1.90)	−6.00	7.25	−1.83 (1.48)	−6.00	3.25	−0.45	<0.01^*∗*^	−0.60	−0.33
J0 (D)	0.17 (0.32)	−0.47	1.60	0.14 (0.35)	−1.49	-1.73	−0.30	<0.01^*∗*^	−0.77	0.02
J45 (D)	−0.01 (0.11)	−0.50	0.40	−0.01 (0.11)	−0.44	0.57	0.01	<0.01^*∗*^	−0.02	0.01

^*∗*^ *P* value <0.05; CI, confidence interval; SD, standard deviation; Min, minimum; Max, maximum; D, diopter; J0, (cylinder/2) cos (2 axis); J45, (cylinder/2) sin (2 axis). ^Δ^Comparison between cycloplegic retinoscopy and 2Win-S (2WIN-S reading minus cycloplegic retinoscopy reading).

**Table 2:** Correlation between cycloplegic retinoscopy and 2WIN-S measurements.

Parameters	Pearson's *r*	95% CI		*P* value
		Lower	Upper	

Sphere (D)	0.876	0.804	0.926	<0.01^*∗*^
Cylinder (D)	0.887	0.750	0.976	<0.01^*∗*^
Spherical equivalent (D)	0.875	0.795	0.928	<0.01^*∗*^

^*∗*^ *P* value <0.05; CI, confidence interval; D, diopter.

**Table 3:** Reliability and repeatability of 2WIN-S.

Parameters	ICC	95% CI		*P* value	Sw
		Lower	Upper		

Spherical equivalent (D)	0.900	0.816	0.939	<0.01^*∗*^	0.61
J0	0.666	0.557	0.748	<0.01^*∗*^	0.30
J45	0.639	0.52	0.728	<0.01^*∗*^	0.31

^*∗*^ *P* value <0.05. CI, confidence interval; D, diopter; ICC, intraclass correlation coefficient; J0, (cylinder/2) cos (2 axis); J45, (cylinder/2) sin (2 axis); Sw,within-subject standard deviation.

## Data Availability

The data used to support the findings of this study are available from the corresponding author upon request.
